# Ligustrazine Attenuates Myocardial Injury Induced by Coronary Microembolization in Rats by Activating the PI3K/Akt Pathway

**DOI:** 10.1155/2019/6791457

**Published:** 2019-05-02

**Authors:** Qiang Su, Xiangwei Lv, Ziliang Ye

**Affiliations:** Department of Cardiology, The Affiliated Hospital of Guilin Medical University, Guilin, Guangxi 541001, China

## Abstract

**Background/Aims:**

Coronary microembolization- (CME-) induced myocardial injury and progressive cardiac dysfunction are mainly caused due to CME-induced myocardial local inflammatory response and myocardial apoptosis. Ligustrazine plays an important protective role in multiple cardiovascular diseases, but its role and the protection mechanism in CME is unclear. This study hypothesized that ligustrazine attenuates CME-induced myocardial injury in rats. This study also explored the mechanism underlying this attenuation.

**Methods:**

Forty SD rats were randomly divided into CME group, ligustrazine group, ligustrazine+LY294002 (ligustrazine+LY) group, and sham group (ten rats in each). In each group, the cardiac function, apoptotic index, serum c-troponin I (cTnI) level, inflammation [interleukin-1*β* (IL-1*β*) and tumor necrosis factor-alpha (TNF-*α*)], and oxidative stress [nitric oxide (NO), superoxide dismutase (SOD), and malondialdehyde (MDA)] were determined. Western blotting was used to detect the proteins which are present in the PI3K/Akt pathway.

**Results:**

Ligustrazine improved cardiac dysfunction induced by CME, increased serum NO and SOD activities, and decreased the serum level in IL-1*β*, MDA, cTnI, and TNF-*α*. Moreover, ligustrazine inhibited myocardial apoptosis, which is perhaps caused by the upregulated Bcl-2, the downregulated cleaved caspase-3 and Bax, and the increased protein level in endothelial nitric oxide synthase and phosphorylated Akt. These effects, however, were reduced if ligustrazine was coadministered with LY294002.

**Conclusions:**

Ligustrazine attenuates CME-induced myocardial injury. The effects associated with this attenuation may be achieved by activating the myocardium PI3K/Akt signaling pathway.

## 1. Introduction

A serious complication in distal microvascular embolism, coronary microembolization (CME), is normally attributed to the detachment of atherosclerotic plaque debris which occurs in percutaneous coronary intervention (PCI). CME can reduce coronary and inotropic reserves and induce no-reflow phenomenon and therefore is considered to be an independent predictor for major cardiac adverse events and poor long-term prognosis [1–4]. Despite reestablishing epicardial coronary vessel patency, primary PCI may fail to restore optimal myocardial reperfusion within the myocardial tissue, a failure at the microvascular level known as no-reflow [5]. The pathophysiology of no-reflow phenomenon is still poorly understood. Proposed mechanisms include distal microembolization of thrombus and plaque debris, ischemic injury, endothelial dysfunction, and individual susceptibility to microvascular dysfunction/obstruction [6–8]. Previous studies have shown that local myocardial inflammation caused by CME is the main cause of progressive cardiac dysfunction [9, 10]. During the development of CME-induced progressive cardiac insufficiency and advanced heart failure, the massive release of inflammatory mediators, for example, IL-1*β* and TNF-*α*, plays a vital role [11, 12]. In addition, several animal studies have shown that the necrotic/apoptotic cardiomyocytes and the microembolic areas occur during the acute phase of CME [13, 14]. In cardiac systolic dysfunction induced by CME, cardiomyocyte apoptosis plays a key role and therefore its suppression reduces CME-induced myocardial injuries [15, 16]. Su et al. [17] reported that cardiomyocyte apoptosis after CME in rats caused myocardial injury. Nicorandil pretreatment significantly reduced CME-induced cardiomyocyte apoptosis and consequently improved myocardial contractile function by activating the PI3K/Akt signaling pathway.

Ligustrazine is an alkaloid monomer which is generally extracted from the rhizome of Chinese medicinal plants that belong to Umbelliferae family, and is an amide alkaloid. It has wide range of pharmacological activities, with high safety and fewer side effects [18]. Ligustrazine has multiple cardiovascular protective effects according to recent studies on cardiovascular diseases, such as antioxidative stress [19], anti-inflammation [20], antiapoptosis [21], antiplatelet aggregation [22], and amelioration of microcirculation [23]. Although the protective effects of ligustrazine on myocardium remained clear, the study by Zhang et al. showed that ligustrazine can still improve the cardiac function in rats with CME [24]. However, the exact mechanism underlying this improvement is unclear. In this condition, an improved understanding of the relationship between ligustrazine and cardiac function in rats with CME is required to aid the therapy and prevention of CME. Therefore, this study investigated the effect of ligustrazine intervention on cardiomyocyte apoptosis, myocardial inflammation, and oxidative stress in CME rats, as well as the PI3K/Akt signaling pathway, which is aimed at clarifying the mechanism underlying the protection of myocardial injury induced by CME from ligustrazine.

## 2. Materials and Methods

### 2.1. Animal Preparation

The Institutional Animal Care and Use Committees at the Guilin Medical University approved all the procedures, which were then carried out as per a protocol on Use of Laboratory Animals which is released from the National Institutes of Health Guidelines. Forty Sprague-Dawley rats (male, weighted 250-300 g) were provided by Guilin Medical University. The rats in the experiment were maintained in humidity- and temperature-controlled houses (50-60% and 23 ± 2°C), with standard laboratory chow and water, in a controlled 12 h/12 h light-dark cycle.

### 2.2. Establishment of the CME Model and Experimental Grouping

Firstly, 30-40 mg/kg pentobarbital was injected intraperitoneally into the rats to keep them under anesthesia. Then tracheotomy was performed, with a ventilator to assist breathing, with the method described by Wang et al. [25]. After this step, thoracotomy was then performed on the left sternal border between the 3^rd^ and 4^th^ intercostal spaces. After this step was finished, the ascending aorta was then clamped for a time of 10 s by a vascular clamp after being separated. Approximately 3000 microspheres (suspended in normal saline of 0.1 mL) with 42 *μ*m diameter (Biosphere Medical Inc., Rockland) were rapidly injected by means of a microinjector from the apex of the left ventricle. After breathing is stabilized, the chest was closed in layers and tracheal intubation was removed. Similarly, saline of 0.1 mL was injected into each rat in the sham group. The 40 SD rats were divided in a random and equal manner into the CME group (*n* = 10), ligustrazine group (*n* = 10), ligustrazine+LY group (*n* = 10), and sham group (*n* = 10). Each rat in the ligustrazine group was administered intragastrically with 27 mg/kg/d ligustrazine (Beijing Yanjing Pharmaceutical Co. Ltd., Beijing, China) for 14 d prior to building the CME model, while in the ligustrazine+LY group, as well as the same administration as the ligustrazine group, each rat was injected intraperitoneally with LY294002 at 30 min prior to building the CME model, at a dose of 10 mg/kg.

### 2.3. Detection of Cardiac Function

At 12 hours following CME, an observation was performed as it was confirmed by Su et al.'s study that it is at this point of time that the lowest cardiac function occurs [26]. Here, a Hewlett Packard Sonos 7500 Ultrasound instrument, with a 12 MHz frequency probe (Philips Technologies, Amsterdam, NY), was applied in this investigation to measure cardiac output (CO), left ventricle fractional shortening (LVFS), left ventricular ejection fraction (LVEF), and left ventricular end-diastolic diameter (LVEDd). In all the measurements, averaged values of triple cardiac cycles were adopted. The echocardiography was conducted by an expert.

### 2.4. Measurement of Serum Cardiac Troponin I (cTnI) Level

At 12 hours following CME or sham operation and before sacrificing, 1.0 mL of blood was collected at the position of femoral vein, and then, serum cTnI level I was determined in line with the kit instructions (Roche Inc., Basel, Switzerland).

### 2.5. Material Collection and Sample Processing

After the detection of cardiac function in the former step, potassium chloride (2 mL,10%) was injected into each rat at the position of the tail vein, for the purpose that the heart of each rat can be harvested immediately while in the ventricular diastolic phase. Atrial appendage and the atria were excluded in the experiment. The ventricle was separated into heart base and the apex at the midpoint of the left ventricle, in a fashion of parallel to the atrioventricular groove. After being processed in liquid nitrogen, the apex was immediately transferred to and preserved at a −80°C refrigerator for the following western blot detection. The base of the heart was embedded using paraffin and then sliced continuously (4 *μ*m for each slice) after being fixed for 12 h with 4% paraformaldehyde. The slices were used for the following staining with hematoxylin-basic fuchsin-picric acid (HBFP) (to observe myocardial microinfarct areas), TdT-mediated dUTP nick-end labeling (TUNEL), and hematoxylin-eosin (HE), which is aimed at observing myocardial microinfarct areas.

### 2.6. Detection of Cardiomyocyte Apoptosis with TUNEL Assay

According to the instructions of the kit (Roche, USA), the apoptotic nuclei was yellow (TUNEL positive) under the light microscope. In each slice (×400 magnification), the number of total cardiomyocytes and apoptotic cardiomyocytes in the microinfarct zone, the infarct zone, and the infarct edge zone were calculated from 40 randomly chosen solitary areas. The apoptosis index (AI) of cardiomyocytes was determined by dividing the apoptotic cardiomyocytes number by the gross cardiomyocytes number ×100% [27].

### 2.7. Measurement of Myocardial Microinfarct Areas

In diagnosing early myocardial ischemia, HBFP staining can stain nucleus in blue color, normal myocardial cytoplasm in yellow, and ischemic myocardium and red blood cells in red. A DMR+Q550 pathological image analyzer (Leica, Wetzlar, Germany) was used to observe( ×100 magnification) each of the HBFP-stained slice. For each slice, five random visual fields were selected. The Leica Qwin analysis software plane was applied in this study to determine the infarct area, which was then divided by gross observed area to calculate the infarct percentage [28].

### 2.8. Antioxidant Enzymes Assay

Commercial kits were used to measure serum SOD, MDA, and NO levels as per the kit's instructions.

### 2.9. Enzyme-Linked Immunosorbent Assay (ELISA) Used in Detecting Inflammatory Cytokines in Serum

Level of TNF-*α* and IL-1*β* in serum was determined by means of an ELISA kit (R&D Systems, Minneapolis, MN) as per the kit's instructions.

### 2.10. Western Blot Analysis

10%-15% SDS-PAGE was used to separate the total protein which was collected in the cardiomyocytes and cardiac tissue before they were electrotransferred to PVDF membrane (Millipore, Atlanta, USA), which were blocked with nonfat milk or 5% bovine serum albumin for 1.5 h at room temperatures before they were incubated at 4°C overnight by using media of primary antibodies against p-Akt, Bcl-2, total Akt, Bax, cleaved caspase-3, or glyceraldehyde-3-phosphate dehydrogenase (GAPDH). All the antibodies were provided by Cell Signaling Technology (Beverly, USA). Secondary antibodies conjugated with horseradish peroxidase were used to incubate the membranes in Tris-buffered saline+Tween 20 (TBST) for 2 h at room temperatures after TBST was used to wash the membranes for 5 times. A chemiluminescence-detecting equipment (enhanced version, Pierce, Holmdel, USA) was used to detect the signals. Image Lab software (Bio-Rad Laboratories, Hercules, CA) was used to assess and quantify the bands for protein amounts.

### 2.11. Statistical Analysis

Statistical analysis was carried out by means of the SPSS 20.0 software (IBM, Chicago, IL). Data were presented in a format of mean value ± standard deviation, and the number of replicates was at least *n* = 3 per group for each data set. Differences were compared by means of the method one-way ANOVA. *P* values < 0.05 were considered statistically significant. GraphPad Prism software version 5.0 (GraphPad Software Inc., San Diego, CA) was used to conduct all of the statistical tests. The sample size for animals or tissue samples in each group is displayed in the legends of figures and table.

## 3. Results

### 3.1. Ligustrazine Improved Cardiac Function after CME

Table 1 shows that cardiac dysfunction was induced by CME, which was characterized by increased left ventricular end-diastolic diameter and decreased cardiac output, left ventricular end-systolic diameter, fractional shortening, and left ventricular ejection fraction. The cardiac dysfunction caused by CME was improved significantly by ligustrazine pretreatment, while LY294002 (a specific inhibitor of the PI3K/Akt signaling pathway) attenuated these protective effects.

### 3.2. Ligustrazine Reduced Serum cTnI Level after CME

**Figure 1:**
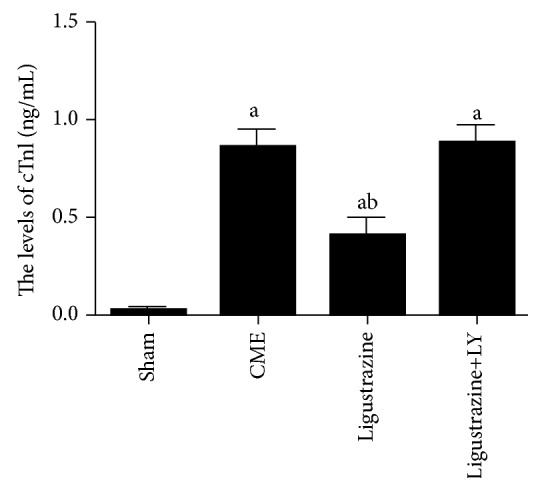
Parameters of cTnI. LY: LY294002; CME: coronary microembolization. ^a^*P* < 0.05 compared to the sham group. ^b^*P* < 0.05 compared to the CME group. *n* = 10.

As shown in Figure 1, in the CME group, serum cTnI levels were significantly enhanced compared to the levels measured in the sham group. On the other hand, ligustrazine significantly inhibited its increase after CME. LY294002 treatment eliminated these effects of ligustrazine, and the levels of cTnI were significantly higher in the ligustrazine+LY group than those in the ligustrazine group.

### 3.3. Effects of Ligustrazine on SOD, MDA, and NO

**Figure 2:**
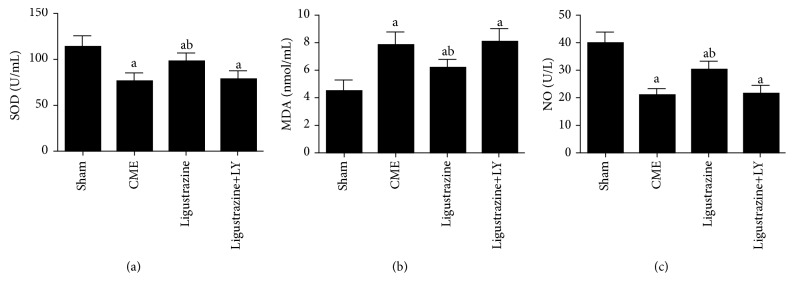
Effect of ligustrazine on serum levels of cardiac oxidative stress parameters [SOD (a), MDA (b), and NO (c)]. CME: coronary microembolization; LY: LY294002. ^a^*P* < 0.05 compared to the sham group. ^b^*P* < 0.05 compared to the CME group. *n* = 10.

Figure 2 indicates that, in comparison to the sham group, the CME group exhibits significantly increased MDA content and significantly decreased SOD and NO level, while these changes were reversed in the ligustrazine group (*P* < 0.05), all of which indicate that, for CME-induced myocardial injury, ligustrazine demonstrates an antioxidative stress effect. However, the effect of ligustrazine on levels of SOD, NO, and MDA is significantly abolished by LY294002. This indicates that ligustrazine's attenuating effect on oxidative stress in CME-induced myocardial injuries is closely related with the PI3K/Akt signaling pathway.

### 3.4. Effect of Ligustrazine on TNF-*α* and IL-1*β*

**Figure 3:**
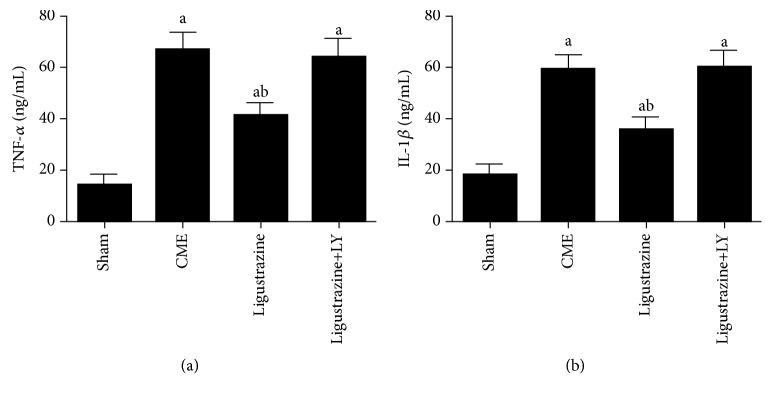
Effect of ligustrazine on TNF-*α* (a) and IL-1*β* (b) in serum. LY: LY294002; CME: coronary microembolization. ^a^*P* < 0.05 compared to the sham group. ^b^*P* < 0.05 compared to the CME group. *n* = 10.

Compared to the sham group, levels of IL-1*β* and TNF-*α* were increased significantly (Figure 3). These levels were inhibited by the administration of ligustrazine. However, the serum IL-1*β* and TNF-*α* concentrations in the ligustrazine+LY group were higher than those in the ligustrazine alone group. These results indicated that ligustrazine can activate the PI3K/Akt signaling. For this reason, the inflammatory cytokine secretion which is induced by CME in the serum is significantly suppressed.

### 3.5. Pathological Observation of CME

**Figure 4:**
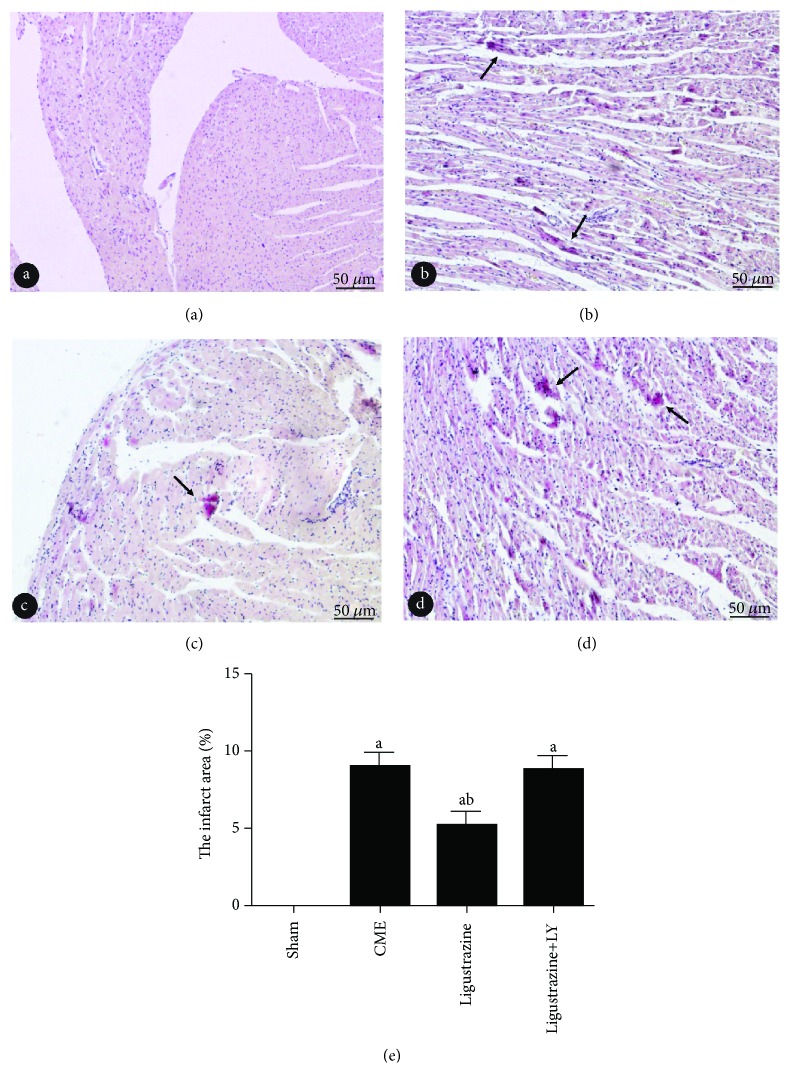
Pathohistological examination by HBFP staining (magnification, ×200; bar = 50 *μ*m). (a–d) Sham group, CME group, ligustrazine group, and ligustrazine+LY group. Ischemic myocardium in red. Arrow indicates microinfarct area. LY: LY294002; CME: coronary microembolization. ^a^*P* < 0.05 compared to the sham group. ^b^*P* < 0.05 compared to the CME group. *n* = 10.

**Figure 5:**
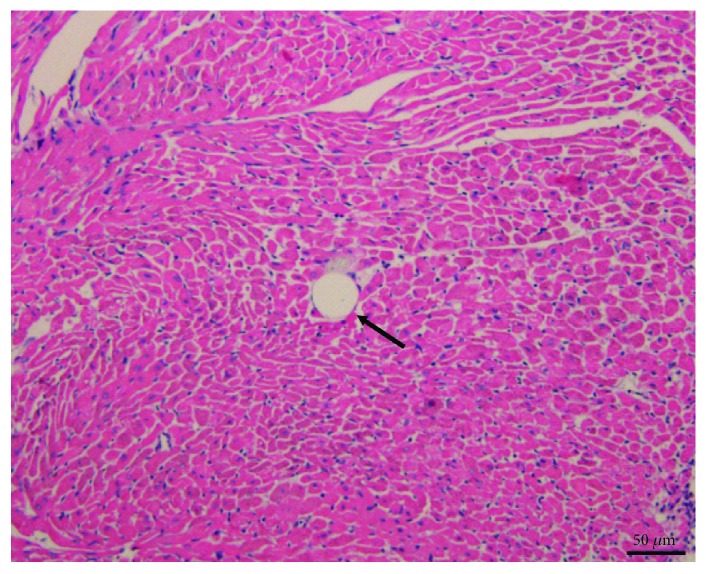
At 12 hours following CME pathohistological examination by HE staining (magnification, ×400; bar = 50 *μ*m). Microspheres with inflammatory cells infiltration are showed in this HE staining. Arrow indicates microspheres. CME: coronary microembolization.

**Figure 6:**
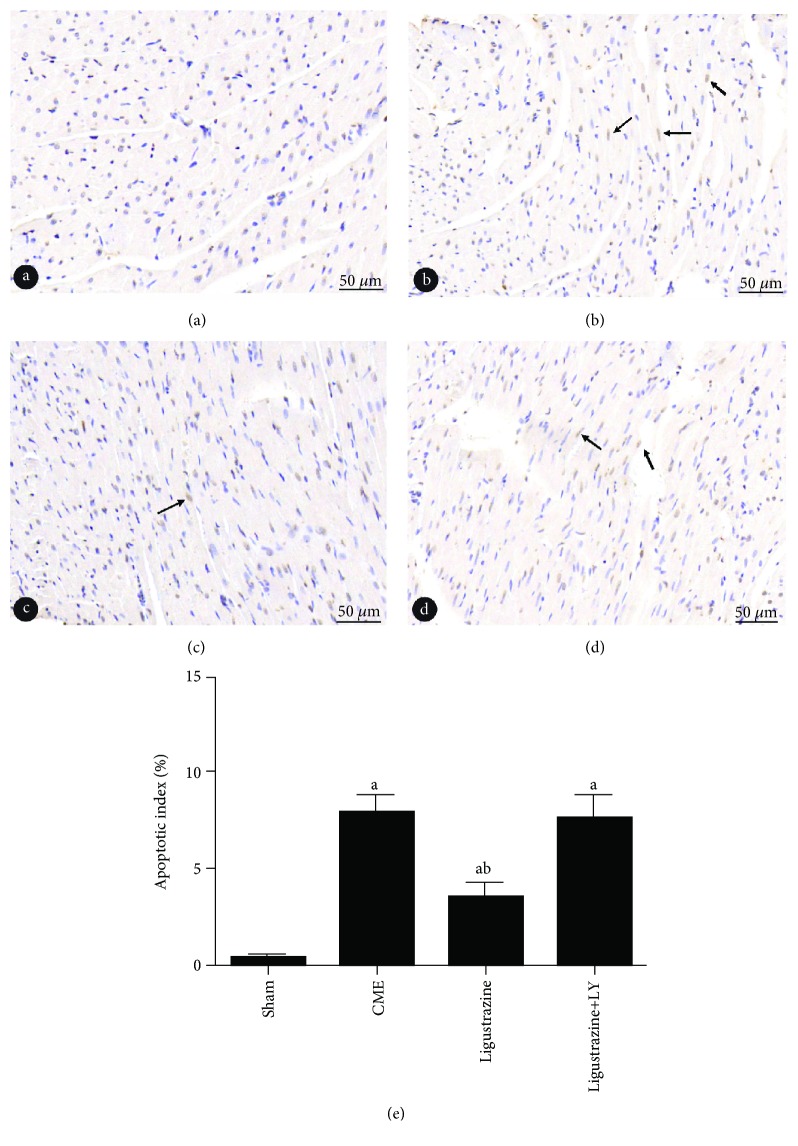
Cardiomyocyte apoptosis is showed with TUNEL staining (magnification, ×400; bar = 50 *μ*m). (a–d) Sham group, CME group, ligustrazine group, and ligustrazine+LY group. Nuclei of apoptotic in yellow while the normal cardiomyocytes in light blue. Arrow indicates nuclei of apoptotic cardiomyocytes. LY: LY294002; CME: coronary microembolization. ^a^*P* < 0.05 compared to the sham group. ^b^*P* < 0.05 compared to the CME group. *n* = 10.

HE and HBFP staining results: albeit no obvious infarcts were noted, subendocardial ischemia occurred occasionally in the sham group. In the other three groups, however, multiple microinfarctions were observed. These lesions were mostly wedge-shaped with a focal distribution and were more common in the subendocardial and left ventricle, as shown in Figure 4. HE staining showed that myocardial cell nucleus dissolved or disappeared in the microinfarction, cytoplasmic red staining, degeneration, peripheral myocardial edema, red blood cell exudation and peripheral inflammatory cell infiltration, and microembolism in arteriole (Figure 5). The infarct size of the CME group, ligustrazine group, and ligustrazine+LY group were (9.17 ± 2.79%), (5.01 ± 1.26%), and (9.03 ± 3.12%), respectively. Compared with the CME group, in the ligustrazine group, the myocardial infarct size was reduced significantly. The apoptotic index of cardiomyocytes was (8.04 ± 1.57%), (3.38 ± 0.63%), (0.39 ± 0.094%), and (7.92 ± 1.65%) in the CME group, ligustrazine group, sham group, and ligustrazine+LY group, respectively (Figure 6). The apoptotic index of cardiomyocytes in the two groups, i.e., the CME group and ligustrazine+LY group, was significantly increased compared with the sham group, while this index was decreased significantly in the ligustrazine group, compared to the CME group.

### 3.6. Ligustrazine Effects on Myocardial Apoptosis

**Figure 7:**
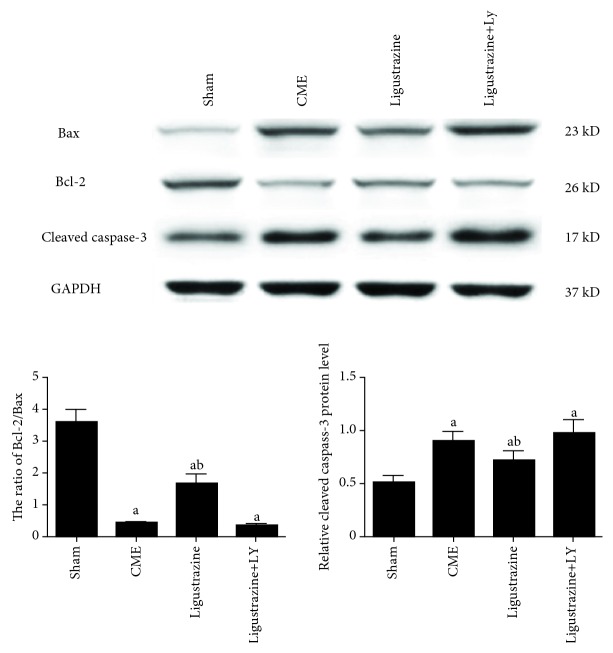
Effect of ligustrazine on myocardial apoptosis. LY: LY294002; CME: coronary microembolization. ^a^*P* < 0.05 compared to the sham group. ^b^*P* < 0.05 compared to the CME group. *n* = 10.

Cleaved caspase-3, Bcl-2, and Bax protein expression was detected to confirm cardiomyocyte apoptosis after CME. Cleaved caspase-3 protein expression in the CME group demonstrated a significant increase compared to the sham group (Figure 7), and the Bcl-2/Bax ratio was downregulated significantly. After pretreatment with ligustrazine, cleaved caspase-3 expression showed significant decrease; the Bcl-2/Bax ratio showed a significant increase. However, if cotreated with LY, the effect of ligustrazine on myocardial apoptosis was significantly attenuated relative to that in the ligustrazine group.

### 3.7. Ligustrazine Effects of the Expression of Proteins on the PI3K/Akt Pathway

**Figure 8:**
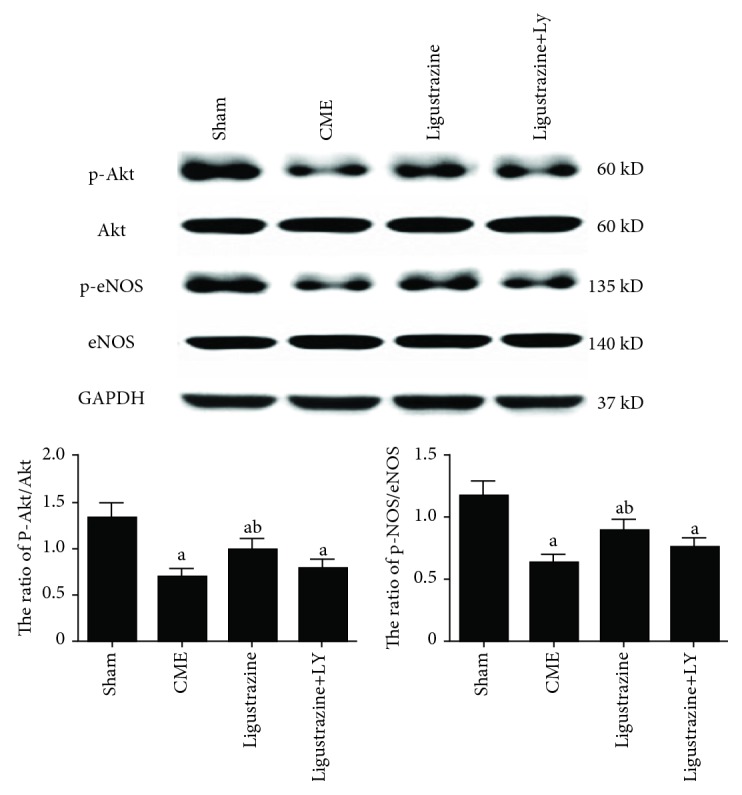
Effect of ligustrazine on the PI3K/Akt signaling pathway. LY: LY294002; CME: coronary microembolization. ^a^*P* < 0.05 compared to the sham group. ^b^*P* < 0.05 compared to the CME group. *n* = 10.

No difference was detected among the four groups regarding the expression of total Akt and eNOS (Figure 8). Myocardial levels of p-Akt and p-eNOS were increased significantly after ligustrazine treatment relative to those in the CME group. However, this upregulation of myocardial p-Akt and p-eNOS induced by ligustrazine was attenuated significantly by LY294002.

## 4. Discussion

The data presented in this study demonstrated that ligustrazine could protect cardiac function in CME rats, which is associated with suppression of the damage and inflammatory cytokines which are triggered by oxidative stress. With these alterations, the cleaved caspase-3 as well as the Bax expression was decreased, the Bcl-2 expression involved in the PI3K/Akt signaling pathway was upregulated, and consequently, the apoptosis after CME was attenuated.

CME often occurs in patients who suffer from unstable plaque ruptures and acute coronary syndrome during PCI. Unlike epicardial proximal vascular occlusion, the CME-induced decrease in left ventricular function has no close connection with the extent of myocardial perfusion defects [29]. This phenomenon cannot be explained by the lack of local myocardial perfusion or microinfarcts. It is now believed that inflammatory reaction and cardiomyocyte apoptosis in normal myocardial tissues around the microinfarction are associated with myocardial injury as well as the progressive cardiac dysfunction after CME [30, 31]. In the abovementioned myocardial injury, however, the PI3K/Akt signaling pathway played a critical role [32]. This study suggests that the levels of serum cTnI, oxidative stress, inflammatory factors, and myocardial apoptosis index are significantly elevated in rats after CME, while at the same time, the cardiac function is deteriorated. Moreover, it is showed here that the PI3K/Akt signaling pathway is downregulated, which indicates that the modeling is successful, in the sense that it is consistent with pathophysiological change in CME.

Ligustrazine is an effective monomer component in the traditional Chinese medicine Ligusticum wallichii [18]. Ligustrazine can resist platelet aggregation, dilate small arteries, improve microcirculation, and promote blood circulation and phlegm [22, 23]. Due to its multiple functions, high safety and diverse mechanisms, it has potential application prospects in cardiovascular diseases [33]. Previous studies have found that ligustrazine pretreatment can reduce MDA levels by 19.2%, increase SOD activity by 39.6%, and reduce oxidative stress in a renal ischemia-reperfusion model of C57BL/6 mice with clamped left renal artery [34]. In a cisplatin-induced rat tubular toxicity model, ligustrazine demonstrates dose-related antiapoptotic and antioxidative effects [35]. In a rat model of cerebral ischemia-reperfusion injury, ligustrazine effectively reduces MDA levels and increased SOD content [36]. Ligustrazine also preserves mitochondrial integrity and mitochondrial function and reduces oxidative brain damage by reducing the production of oxygen free radical, suggesting that ligustrazine has an antioxidative effect [37]. The data presented in this study shows that ligustrazine in a rat CME model can effectively inhibit myocardial inflammation, reduce oxidative stress level, scavenge oxygen free radicals, reduce lipid peroxidation, and protect mitochondrial structure and function, thereby reducing myocardial injury induced by CME.

In recent years, the PI3K/Akt signaling pathway has been the focus for reducing CME-induced myocardial injury, as a common pathway for many drugs to achieve myocardial protection [17, 32]. A variety of intracellular signal transduction mediators and effector proteins are involved in the PI3K/Akt signaling pathway, wherein eNOS is one of the downstream targets of the signaling pathways [38]. Activated Akt promotes phosphorylation of serine 1177 at eNOS, thereby upregulating eNOS expression, ultimately reducing the mitochondrial permeability transition pore opening, maintaining mitochondrial outer membrane stability, and improving mitochondrial energy production. This in turn reduces apoptosis and protects the myocardium [39]. In this study, ligustrazine upregulates the expression of eNOS phosphorylation, while the PI3K/Akt signaling pathway inhibitor LY294002 abolishes the phosphorylation of eNOS induced by ligustrazine, indicating that the activation of eNOS by ligustrazine is produced by the PI3K/Akt signaling pathway. However, serum cTnI, inflammatory cytokines, oxidative stress, myocardial apoptosis, and cardiac function in the ligustrazine+LY group show no significant differences from those in the CME group, suggesting that LY294002 can attenuate myocardial protection of ligustrazine. Therefore, this study demonstrates that the PI3K/Akt signaling pathway is closely associated with the protective effects of ligustrazine on myocardial injury. Ligustrazine may exert myocardial protection by activating the PI3K/Akt signaling pathway and the downstream eNOS and by sequentially activating the downstream targets.

## 5. Conclusions

In summary, ligustrazine has a definite effect on anti-CME-induced myocardial injury. This effect may be due to the reduction of myocardial apoptosis, oxidative stress, and the myocardial inflammation through the PI3K/Akt signaling pathway activation. The data presented in this study can be used as a theoretical basis in applying ligustrazine in the therapy and prevention of myocardial injury induced by CME.

## Figures and Tables

**Table 1:** Changes in cardiac function (*x* ± *s*).

Group	*n*	LVEF (%)	LVFS (%)	CO (L/min)	LVEDd (mm)
Sham	10	80.73 ± 5.56	44.03 ± 4.64	0.190 ± 0.032	5.08 ± 0.45
CME	10	58.68 ± 3.77^a^	21.79 ± 2.72^a^	0.109 ± 0.008^a^	7.84 ± 0.59^a^
Ligustrazine	10	69.23 ± 3.99^ab^	37.81 ± 4.95^ab^	0.169 ± 0.018^ab^	6.62 ± 0.52^ab^
Ligustrazine+LY	10	56.52 ± 3.64^a^	20.88 ± 2.76^a^	0.106 ± 0.009^a^	7.89 ± 0.65^a^

CME: coronary microembolization; LVFS: left ventricle fractional shortening; LVEDd: left ventricular end-diastolic diameter; CO: cardiac output; LY: LY294002; LVEF: left ventricle ejection fraction. ^a^*P* < 0.05 compared with the sham group. ^b^*P* < 0.05 compared with the CME group.

## Data Availability

The data used to support the findings of this study are available from the corresponding author upon request.
